# The Effect of Upper Extremity Fatigue on Grip Strength and Passing Accuracy in Junior Basketball Players

**DOI:** 10.2478/hukin-2013-0027

**Published:** 2013-07-05

**Authors:** Taghread Ahmed

**Affiliations:** 1 Faculty of physical education, Zagazig University, Egypt.

**Keywords:** muscle fatigue, exercise intensity, hand grip strength, upper extremity, passing accuracy, basketball

## Abstract

Fatigue is an unavoidable part of a basketball game, which may affect an athlete’s performance. The aim of this study was to investigate the effect of upper extremity fatigue on grip strength and passing accuracy in basketball, and ascertain if the effects of different fatigue protocols on grip strength and passing accuracy are the same. Twenty-four juniors under 18 years old (age: 16.75 ± 0.62 years; body height: 184.5 ± 3.31 cm; body mass: 77.25 ± 3.22 kg) volunteered to participate in the study, and were divided into two groups. After a warm-up, both groups performed the basketball passing test and grip strength was recorded for each group under three different testing conditions: rest, 70% and 90% exercise intensity. The protocol used for the first group was the chest press, and for the second group the wrist curls. Results show that after the upper extremity fatigue protocol all parameters of the study (grip strength and passing accuracy) showed a significant decrease, and there was no significant difference between both groups regarding grip strength and passing accuracy. The study suggested that in order to avoid upper extremity fatigue, basketball trainers and coaches need to include upper extremity conditioning exercises into their training sessions.

## Introduction

Basketball is a game of continuously changing tempo, requiring speed, acceleration, explosive movements such as rebounding, passing, jump shooting, fast breaks and high speed play. The game also involves skills that must be applied dynamically, explosively and repeatedly ( Gore, 2000 ). Handgrip strength is important in basketball as various movements rely on the continuous use of wrist and digit flexors in catching, holding, shooting and throwing the ball ( Cortis et al., 2011 ; Visnapuu et al., 2007).

Upper extremity muscle strength and grip strength are the primary factors affecting passing accuracy. Grip strength is correlated with the strength of the upper extremity, general strength of the body and some anthropometric measurements ( Balogun et al., 1991 ). Grip power is the result of forceful flexion of all finger joints with maximum voluntary force that the subject is able to exert under normal biokinetic conditions ( Koley et al., 2009 ). The synergistic action of flexor and extensor muscles and the interplay of muscle groups is an important factor in the strength of the resulting grip. Many factors influence grip strength, including general muscle strength, hand dominance, fatigue, time of day, age, nutritional status, restricted motion, and pain ( Richards et al., 1996 ).

Muscle fatigue occurs with prolonged or repetitive use of a muscle group. The mechanism of fatigue is multifactorial and not fully understood, but it involves the central nervous system, peripheral nervous system, muscle units and individual muscle fibers. At the level of muscle cells, depletion of energy stores may be a significant factor ( Enoka and Duchateau, 2008 ). Fatigue is a very complex concept, involving both psychological and a host of physiological factors ( Astrand and Rodahl, 2003). Consequently fatigue should never be viewed as a single entity or process. Rather it is a highly complex phenomenon comprising of numerous different components and acting at multiple sites within both the central nervous system and the muscle cells ( McKenna and Hargreaves, 2008 ).

Fatigue is especially important in a sporting context and in a team game such as basketball, fatigue may be the determining factor between winning and losing. The study of fatigue relative to performance of different skills has long been a subject of practical and scientific interest to strength and conditioning professionals, trainers, coaches and sport scientists. Research to date however, has provided conflicting and often contradictory findings, partly due to the inconsistent experimental designs and procedures used. Anshel and Novak (1989) partly attribute the conflicting results to poor control of the participant’s fitness or strength levels and the intensity of administered fatigue. This picture is made even more complex by the fact that fatigue is difficult to define. Previous investigations examining the effect of fatigue on performance in basketball are very scarce indeed. However, Ivoilov et al. (1981) examined the effects of a two versus two game of basketball on shooting performance and found that basketball-shooting accuracy deteriorated significantly following the fatigue protocol. McMorris et al. (1994) examined passing performance following rest, exercise at 70%, and 100% of maximum power output. Results showed that for total points scored, performance following exercise at 70% maximum power output was significantly (p < 0.01) better than in the other two conditions, which did not differ significantly. Uygur et al. (2010) examined the effects of fatigue on the kinematics of free throw shooting in basketball. The results demonstrated that fatigue did not affect free throw shooting kinematics (p>0.05), and there was no significant joint angle difference between successful and unsuccessful shots (p>0.05). Lyons et al. (2006) examined the impact of moderate and high intensity total body fatigue on passing accuracy in elite and novice basketball players. On the examination of the mean scores, according to them, it was clear that high intensity exercises caused significant detriment in passing performance of both novice and expert basketball players when compared to their resting scores. Therefore, the detrimental impact of fatigue on passing performance was not as steep in elite players as compared to novice ones.

According to the best of the researcher's knowledge, there is no study that has examined the effect of upper extremity fatigue on grip strength, and passing accuracy in basketball. Therefore, we decided to conduct this research because of the importance of grip strength and passing accuracy in basketball, which will seek to contribute to the lack of scientific information, Consequently the two main aims of this study were (1) to investigate the effects of upper extremity fatigue on grip strength and passing accuracy in basketball, and (2) to ascertain if the effects of different fatigue protocols on grip strength and passing accuracy have the same effect.

## Material and Methods

### Participants

Twenty-four male junior basketball players from 2 teams (age: 16.75 ± 0.62 years; body height: 184.5 ± 3.31 cm; body mass: 77.25 ± 3.22 kg;) volunteered to participate in this study. They were divided into two equally sized experimental groups. Before applying the fatigue protocols the researcher compared the two experimental groups in pre-test for grip strength and passing accuracy. No significant differences between the two groups were observed.

### Measures

Body height and body mass were measured with subjects in training clothes and barefoot. Body height was measured using a stadiometer, accurate to within 1 cm (SECA, mod 213, Germany). Body mass was measured using a digital medical scale, accurate to within 0.1 kg (SECA, mod 769, Germany).

### Grip strength testing

A portable digital hand dynamometer (Jamar, EN-120604) was used to measure grip strength. Participants were evaluated in a standing position, arms at their side, not touching their body, elbows bent slightly. The participants were asked to squeeze the dynamometer three times with each hand. There was a one minute rest period between each trial in order to avoid the effects of muscle fatigue. The result of each trial was recorded to the nearest kilogram. The mean value of three squeezes was taken into account ( Ross and Rosblad, 2002 ).

### The AAHPERD Basketball Passing Test

This test was chosen because it is an appropriate test for assessing basketball passing skills. The test was validated by the American Alliance for Health, Physical Education, Recreation and Dance in 1984 using senior high school students. The test-retest approach computed reliability coefficients of 0.84 to 0.97 so the test is both valid and reliable.

The test also required participants to pass the ball quickly and accurately, two elements fundamental to passing in basketball ( Krause et al., 1999 ). The test required a smooth wall surface of 30 feet. A restraining line 26 feet long was marked out on the floor 8 feet from and parallel to the testing wall. On the testing wall six boxes measuring 2 feet by 2 feet were marked out all 2 feet apart. Moving from the left side of the testing wall, targets A, C and E have their base 5 feet from the floor while B, D and F have their base 3 feet from the floor.

The player stood behind the 8-foot restraining line, holding a basketball and facing the far left wall target (A). Then, an assistant played the CD, which emitted a three-beep countdown, and the fourth beep signaled the start of the test. Following the fourth beep, each player performed a chest pass to the first target square (A), recovered the ball while moving to the second target square (B), performed a chest pass to the second target (B). The player then continued this action until he reached the last target (F). While at the last target (F), they threw two chest passes then repeated the sequence by moving to the left, passing at targets E, D, C and so on. The only modification to the test was that it continued for just thirty seconds. Only chest passes were allowed. The scoring of the test was as follows:

- Two points were awarded for each chest pass that hit the target or on the target lines.
- One point was awarded for every pass that hit between the targets.
- No point was awarded if a player’s foot was on or over the restraining line, or if a pass other than a chest pass was used.

The test result was obtained by totaling all the points scored over 30 s.

### Procedures

#### Experimental design

**Figure 1:**
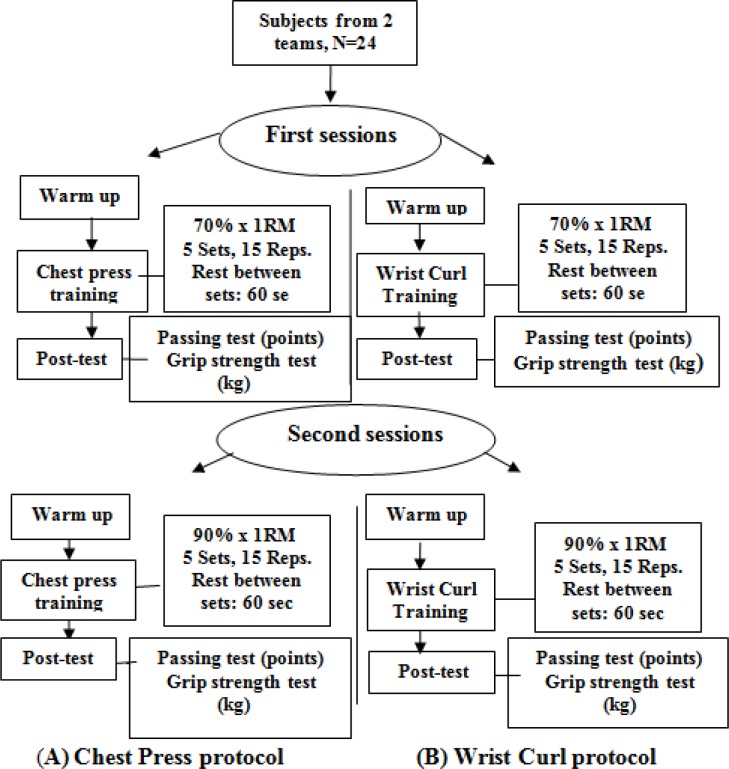
*
Experimental protocols with chest press (A) and wrist curl (B) training
*

Figure 1 outlines the experimental procedure of this study. Before the study, participants had to perform a 1 RM (Repetition of Maximum) test for the chest press and wrist curls to determine the fatigue protocol intensity. These were established by calculating 70% and 90% of the maximum number of chest press and wrist curls performance within a minute. It enabled the researcher to establish fatigue intensities based on the fitness level of each individual, and ensured that each participant was working with the same intensity. Grip strength was recorded with a hand dynamometer, the basketball passing test used in this study was based on a modification of the AAHPERD (1984) basketball passing test. Each participant was given one attempt on the test to familiarize himself with the protocol. Participants were then given a 5–10 minute warm up prior to their performance under fatigue conditions. After that, one team performed the chest press protocol (A) and the other one performed the wrist curl protocol (B). Each group was fatigued in two separate sessions, one week apart, after determining 1 RM and recorded grip strength and passing accuracy. In the first session the two experimental groups trained using exercise intensity of 70% maximal repetitions within a minute, and after one week both groups performed the second session, using exercise intensity of 90% maximal repetitions within a minute. A single-group, pre-test, post-test repeated measures design was used. Upper-extremity muscle fatigue was the independent variable with two exercise intensities (70% – 90%) of 1 RM. The dependent variables were grip strength and passing accuracy.

#### Dumbbell chest press protocol

Participants lay flat on a bench with feet on the floor, arms extended upward, holding dumbbells with an overhand grip. They lowered the dumbbells to the chest level, bending elbows and rotating forearms to bring their hands into pronation (palms facing legs), and exhaled to press dumbbells back up until arms were straight. After that, they slowly lowered the weight in a controlled fashion down to starting position. They repeated the movement for a desired number of repetitions. During the exercise protocol, each participant kept spine in a neutral position, and kept the head on the bench. The muscles engaged in this exercise were chest, shoulders and triceps ( Pearl, 2005 ).

### Statistical Analysis

All analyses were executed in SPSS for windows version 16.0 (SPSS Inc., Chicago, IL). Values were expressed as mean and standard deviation (SD). The independent samples t-test was used to determine the mean differences in grip strength and passing accuracy between the two experimental groups following upper extremity fatigue protocols. The paired samples t-test was used to compare the effect of upper extremity fatigue on grip strength and passing accuracy for each group. The level of significance was set at *p* < 0.05.

## Results

**Figure 2:**
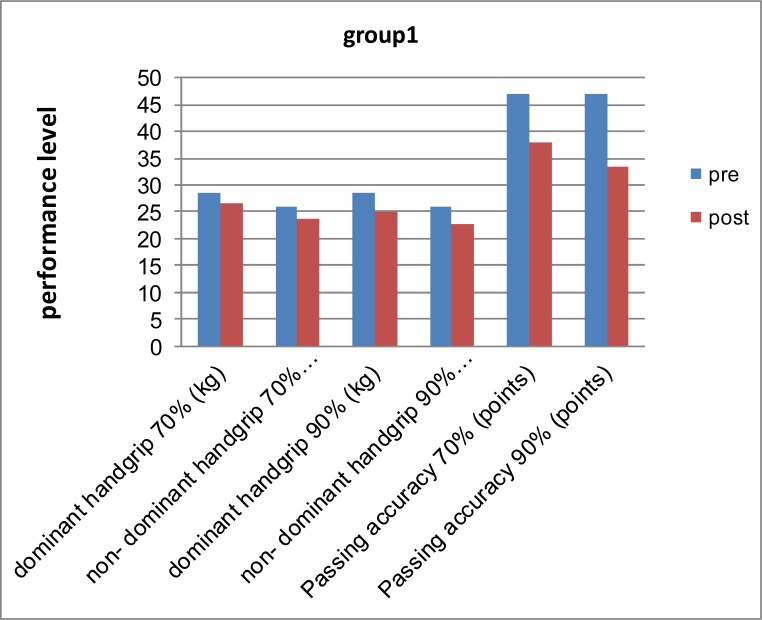
*
Differences between pre and post measurements for group 1.
*

Table 1 shows the results following the fatigue protocol (Dumbbell Chest Press). It is clear that the performance of group 1 in the dominant hand grip declines from (28.41) at rest to (26.75 points) following moderate intensity exercise (70% of RM), and from (28.41) at rest to (25 points) following high intensity exercise (90% of RM). The performance of the non-dominant hand grip declines from (25.83) at rest to (23.75 points) following moderate intensity exercise (70% of RM), and from (25.83) at rest to (22.58 points) following high intensity exercise (90% of RM). The performance of passing accuracy declines from (46.91) at rest to (37.83 points) following moderate intensity exercise (70% of RM), and from (46.91) at rest to (33.25 points) following high intensity exercise (90% of RM). All parameters of the study, grip strength and passing accuracy showed a significant decrease (p<0.05). Figure 2 shows the differences between pre and post measurements for group 1.

**Figure 3:**
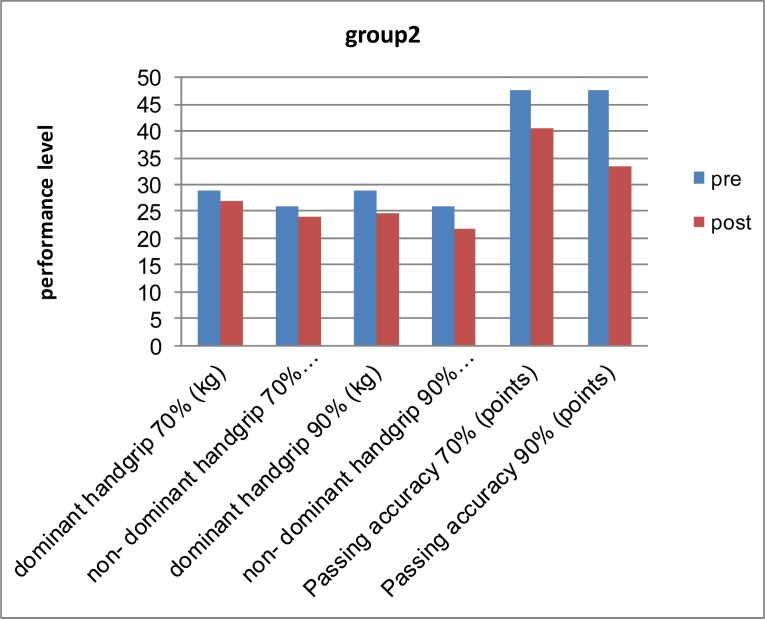
*
Differences between pre and post measurements for group 2.
*

Table 2 shows the results following fatigue protocol (Dumbbell Wrist Curls). It is clear that the performance of group 2 in the dominant hand grip declines from (28.75) at rest to (26.91 points) following moderate intensity exercise (70% of RM), and from (28.75) at rest to (24.66 points) following high intensity exercise (90% of RM). The performance of the non-dominant hand grip declines from (26.08) at rest to (24 points) following moderate intensity exercise (70% of RM), and from (26.08) at rest to (21.91 points) following high intensity exercise (90% of RM). The performance of passing accuracy declines from (47.58) at rest to (40.5 points) following moderate intensity exercise (70% of RM), and from (47.58) at rest to (33.41 points) following high intensity exercise (90% of RM). All parameters of the study, grip strength and passing accuracy showed a significant decrease (p<0.05). Figure 3 shows the differences between pre and post measurements for the group 2. After applying the fatigue protocols the researcher compared between the two experimental groups in post-test for grip strength and passing accuracy by using post-test mean, standard deviations (SD) and significant differences. Table 3 shows a significant difference in the post measurements between the two experimental groups. The results showed no significant difference (p>0.05) in the performance between both groups regarding the grip strength and passing accuracy following fatigue protocols at exercise intensity (70% of 1 RM), and fatigue protocols at exercise intensity (90% of 1 RM).

## Discussion

The present study was conducted to investigate the effect of upper extremity fatigue on grip strength and passing accuracy in basketball, and to ascertain if the effects of different fatigue protocols on grip strength and passing accuracy are similar. The results from this study showed that there were significant differences (p<0.05) in the performance between pre-test, post-test for group 1 which used the dumbbell chest press as a fatigue protocol. The paired samples t-test was used to examine the mean difference in the performance at rest and the performance under the influence of exercise intensity of 70% of RM in the dominant hand grip, non-dominant hand grip and passing accuracy for group 1. It showed that there was a significant difference (p<0.05) in the results between the performance at rest and the performance following moderate intensity exercise. The same t-test was used to examine the mean difference in the performance at rest and the performance under the influence of exercise at intensity of 90% of RM, in the dominant hand grip, non-dominant hand grip and passing accuracy. The results showed that there was a significant difference (p<0.05) in the performance between the performance at rest and that following high intensity exercise. Figure 2 shows that there was a clear deterioration in the performance for group 1, which used the dumbbell chest press fatigue protocols.

The paired samples t-test was used to examine the rate of decline or the change in the dominant hand grip, non-dominant hand grip and passing accuracy for group 2 from the rest to exercise of intensity 70% of RM. Results showed that there was a significant difference (p<0.05) in the performance between the performance at rest and that following moderate intensity exercise. The same t-test was used to examine the mean difference in the performance at rest and the performance under the influence of exercise at intensity of 90% of RM, in the dominant hand grip, non dominant hand grip and passing accuracy. Results showed that there was a significant difference (p<0.05) in the results between the performance at rest and that following high intensity exercise. Figure 3 shows that there is a clear deterioration in the performance for group 2, which used the dumbbell wrist curls fatigue protocols. The researcher believes that these differences between the means of pre and post measurements at p < 0.05 for the two groups are due to the effect of upper extremity fatigue on grip strength and passing accuracy in basketball. Both groups had difficulty in maintaining the balance and postural stability immediately following fatigue conditions. Muscle fatigue caused by repeated muscle contractions reduced neuromuscular coordination and it would be more difficult to control the passing angle and power output. Muscle strength is one of the factors that affect the strength of the grip. The synergistic action of flexor and extensor muscles and the interplay of muscle groups is an important factor in the strength of resulting grip ( Richards et al., 1996 ). This detrimental effect of fatigue on sports related performance and skills have been reported in the literature. It was found that fatigue had an adverse effect on passing accuracy in basketball and this effect was more prominent in novice players when compared to elite ones ( Lyons et al., 2006 ). Ivoilov et al. (1981) found that basketball-shooting accuracy deteriorated significantly following a fatigue protocol.

The independent sample t-test was used to test the difference in performance at exercise intensity of 70% of 1 RM between the two groups in the dominant hand grip, non-dominant hand grip and passing accuracy. The same t-test was used to test the difference in performance at exercise of intensity of 90% of 1 RM between the two groups in the same variables. Results showed no significant difference (p>0.05) in the performance between both groups. This means that the two fatigue protocols which were used had the same effect on grip strength and passing accuracy in basketball. Upper extremity muscle strength and grip strength is one of the factors affecting the accuracy of the passing and the grip strength is correlated with the strength of the upper extremity ( Balogun et al., 1991 ). From the above results, it may be concluded that fatigue of the muscles of the upper extremity adversely affects grip strength and consequently the accuracy of passing.

Trying to identify the physiological mechanisms underlying fatigue effects on performance in this study is both challenging and highly complex. Additionally, mechanisms of fatigue are still not understood ( Lee et al., 2000 ). The fatiguing task impacted heavily on a number of major muscle groups in the lower body. It is likely therefore, that muscle glycogen degradation in large muscle groups, which were then subsequently used in the passing task, was one causative factor.

## Conclusions and Practical Implications

The major conclusion drawn from this study according to the results was that upper extremity fatigue with exercise intensity of 70% of 1 RM, and exercise intensity of 90% of 1 RM, had a significantly negative effect on grip strength, and passing accuracy in junior basketball players. There was no significant difference between the two groups in post-test for grip strength and passing accuracy. This means the two fatigue protocols used had a similar effect. This indicates that trainers and coaches in basketball should avoid upper extremity fatigue, which may affect the level of performance. Therefore, they need to include upper extremity exercise into their training sessions. In the future it would be noteworthy to study whether and how fatigue impacts on some other skills in basketball, which were not discussed in this study. Most of all, it would be interesting to find out whether fatigue causes quantitative and qualitative changes in the passing technique, and to analyse these changes.

## Figures and Tables

**Table 1:** *
Means, standard deviations (SD) and significant differences between pre and post measurement for group (1) on handgrip strength and passing accuracy.
*

** Variables **	** Pre-measurement **		** Post-measurement **		** Difference between means **	** T **	** P **

	** Mean **	** SD± **	** Mean **	** SD± **			
** Dominant handgrip 70% (kg) **	28.41	1.72	26.75	1.48	1.66	11.72	0.01
** non-Dominant handgrip 70% (kg) **	25.83	1.33	23.75	1.05	2.08	9.1	0.01
** Dominant handgrip 90% (kg) **	28.41	1.72	25	1.41	3.41	14.92	0.01
** non-Dominant handgrip 90%(kg) **	25.83	1.33	22.58	1.16	3.25	14.93	0.01
** passing accuracy 70% (points) **	46.91	4.58	37.83	4.42	9.08	11.6	0.01
** Passing accuracy 90% (points) **	46.91	4.58	33.25	3.13	13.66	13.53	0.01

**Table 2:** *
Means, standard deviations (SD) and significant differences between pre and post measurement for group (2) on handgrip strength and passing accuracy.
*

** Variables **	** Pre-measurement **		** Post-measurement **		** Difference between means **	** T **	** P **

	Mean	SD±	Mean	SD±			
** Dominant handgrip 70% (kg) **	28.75	1.42	26.91	1.37	1.83	5.69	0.01
** non- Dominant handgrip 70% (kg) **	26.08	1.08	24	0.73	2.08	8.01	0.01
** Dominant handgrip 90% (kg) **	28.75	1.42	24.66	1.43	4.08	12.14	0.01
** non- Dominant handgrip 90% (kg) **	26.08	1.08	21.91	1.31	4.16	11.38	0.01
** Passing accuracy 70% (points) **	47.58	4.77	40.5	6.77	7.08	6.37	0.01
** Passing accuracy 90% (points) **	47.58	4.77	33.41	5.8	14.16	16.64	0.01

**Table 3:** *
Means, standard deviations (SD) and significant differences in the post measurement between experimental groups on handgrip strength and passing accuracy.
*

** Variables **	** Group 1 **		** Group 2 **		** Difference between means **	** T **	** P **

	Mean	SD±	Mean	SD±			
** Dominant handgrip 70% (kg) **	26.75	1.48	26.91	1.37	−0.16	−0.285	0.77
** non- Dominant handgrip 70% (kg) **	23.75	1.05	24	0.73	−0.25	−0.672	0.50
** Dominant handgrip 90% (kg) **	25	1.41	24.66	1.43	0.33	0.573	0.57
** non- Dominant handgrip 90% (kg) **	22.58	1.16	21.91	1.31	0.66	1.31	0.20
** Passing accuracy 70% (points) **	37.83	4.42	40.5	6.77	−2.66	−1.14	0.26
** Passing accuracy 90% (points) **	33.25	3.13	33.41	5.8	0.16	−0.08	0.93
